# Variation in biosynthesis of an effective anticancer secondary metabolite, mahanine in *Murraya koenigii*, conditional on soil physicochemistry and weather suitability

**DOI:** 10.1038/s41598-020-77113-y

**Published:** 2020-11-18

**Authors:** Raghuram Kandimalla, Momita Das, Sagar R. Barge, Partha Pratim Sarma, Dibya Jyoti Koiri, Arundhuti Devi, Arjun Kumar Karki, Anil Kumar, Rajlakshmi Devi, Bikas C. Pal, Narayan C. Talukdar, Suman Kumar Samanta

**Affiliations:** 1 Drug Discovery Laboratory, Life Sciences Division, Institute of Advanced Study in Science and Technology, Vigyan Path, Paschim Boragaon, Guwahati, Assam 781035 India; 2 Environmental Chemistry Laboratory, Resource Management and Environment Section, Life Sciences Division, Institute of Advanced Study in Science and Technology, Vigyan Path, Paschim Boragaon, Guwahati, Assam 781035 India; 3 Present Address: James Graham Brown Cancer Center, University of Louisville, Louisville, KY 40202 USA; 4 Present Address: Assam Down Town University, Panikhaiti, Guwahati, Assam 781006 India

**Keywords:** Chemical biology, Drug discovery, Plant sciences

## Abstract

*Murraya koenigii* (MK) leaf being a rich source of bioactive secondary metabolites has received inordinate attention in drug development research. Formation of secondary plant metabolite(s) in medicinal plants depends on several factors and in this study the cause of variation in bioavailability and content of a vital bioactive phytochemical, mahanine in the MK leaves from different geographical locations of varying soil properties and weather parameters was determined. Accordingly, MK leaves and soil samples around the plant base in quintuplicate from each site across five states of India at similar time point were collected. Mahanine content was determined and compared among samples from different regions. The quantitative analysis data comprised that MK-leaves of southern part of India contains highest amount of mahanine, which is 16.9 times higher than that of MK-leaves of north-eastern part of India (which measured as the lowest). The results suggested that pH, conductivity and bacterial populations of the soil samples were positively correlated with mahanine content in the MK-leaves. For examples, the average soil pH of the southern India sites was in basic range (8.8 ± 0.6); whereas that of the north-east India sites was in slightly acidic ranges (6.1 ± 0.5) and mean soil conductivity value for the north east India soils was 78.3 ± 16.3 µS/cm against mean value of 432.4 ± 204.5 µs/cm for south India soils. In conclusion, this study proclaims that higher level of bioactive phytochemical, mahanine in MK leaves depending upon geographical location, weather suitability and soil’s physiochemical and microbial parameters of its cultivation sites.

## Introduction

*Murraya koenigii* (MK) (L.) Spreng is a medicinal plant of high value which generally grown profusely in the tropical and subtropical region of Asia^1^. Secondary metabolites in MK plant leaf are known to exhibit diverse medicinal properties including anti-cancer, anti-diabetics, anti-arthritics activity^2,3^. The MK plant leaf is very popular and has been used in the Indian cuisine for its characteristic aroma^4^. The chemical profiling of MK leaves suggests that the secondary metabolites in MK leaf are several alkaloids, terpenoids, flavonoids and phenolic^5^. Few isolated pure compounds from the MK leaf have been studied thoroughly for their details signal cross talk mechanism in different disease models including cancer^6^. For example, mahanine, a carbazole alkaloid isolated from MK leaves established its ability to combat against diabetes and the different types of cancer^7,8^. In a recent review^9^, we discussed in details the molecular mechanistic insight of the mahanine in different cancer model. In another study, we established mahanine as potent chemotherapeutic option for different subtypes of breast cancer^2^.

The naturally occurring secondary metabolite of a plant system has been shown to defend the exogenous influences such as weather, microbes, bacteria etc.^10^. The direct role of secondary metabolites on plant growth and maturity is still not clear and needs further thorough research^11,12^, although the contribution of that secondary plant metabolite (SPM) towards pharmaceutical drug development has been well recognized for centuries^13^. The list of examples includes taxol, vincristine, penicillin and vinblastine as leading drugs in the market from natural sources^14^. Several other SPM such as ingenol, curcumin, mebutate, betulinic acid, withaferin-A are currently in clinical trials and likely to emerge as effective chemotherapeutic agents in market^15,16^. The combination of chemistry and biology found in nature, is much easier to understand than the laboratory chemistry; and hence it always gives a decent solution as drug for diseases management^17^. Natural products with anti-tumor activity belong to several structurally diverse groups and hence it is difficult to make their classification in a single group^18^. Mechanism of formation and accumulation of those specific toxic material as secondary metabolites in the plant parts is quite ambiguous. In one of our recent study^2^, which involved isolation procedure of mahanine from MK leaves, we witnessed varied range of biosynthesis (of mahanine) depending upon the place, time of collection of the leaf samples as well as weather conditions of the plant sample collection sites. Recently a group of researcher showed that, the seasonal variation of carbazole alkaloids distribution in MK leaves and thereafter emphasized on the potentiality of the EtOAc extract against the cancer treatment^19^. The present study was carried out to comprehend the matrixes behind the formation of such type of variation of secondary metabolite in the plant parts. The primary objectives of this study were to (i) determine the variation of mahanine in MK leaves collected at single time point from different geographical locations of varying soil and weather parameters and (ii) possible association of soil, microbes and weather parameters with the mahanine biosynthesis in MK leaves of diverse locations.

## Methods and materials

### Selection of sites and collection of samples

**Figure 1:**
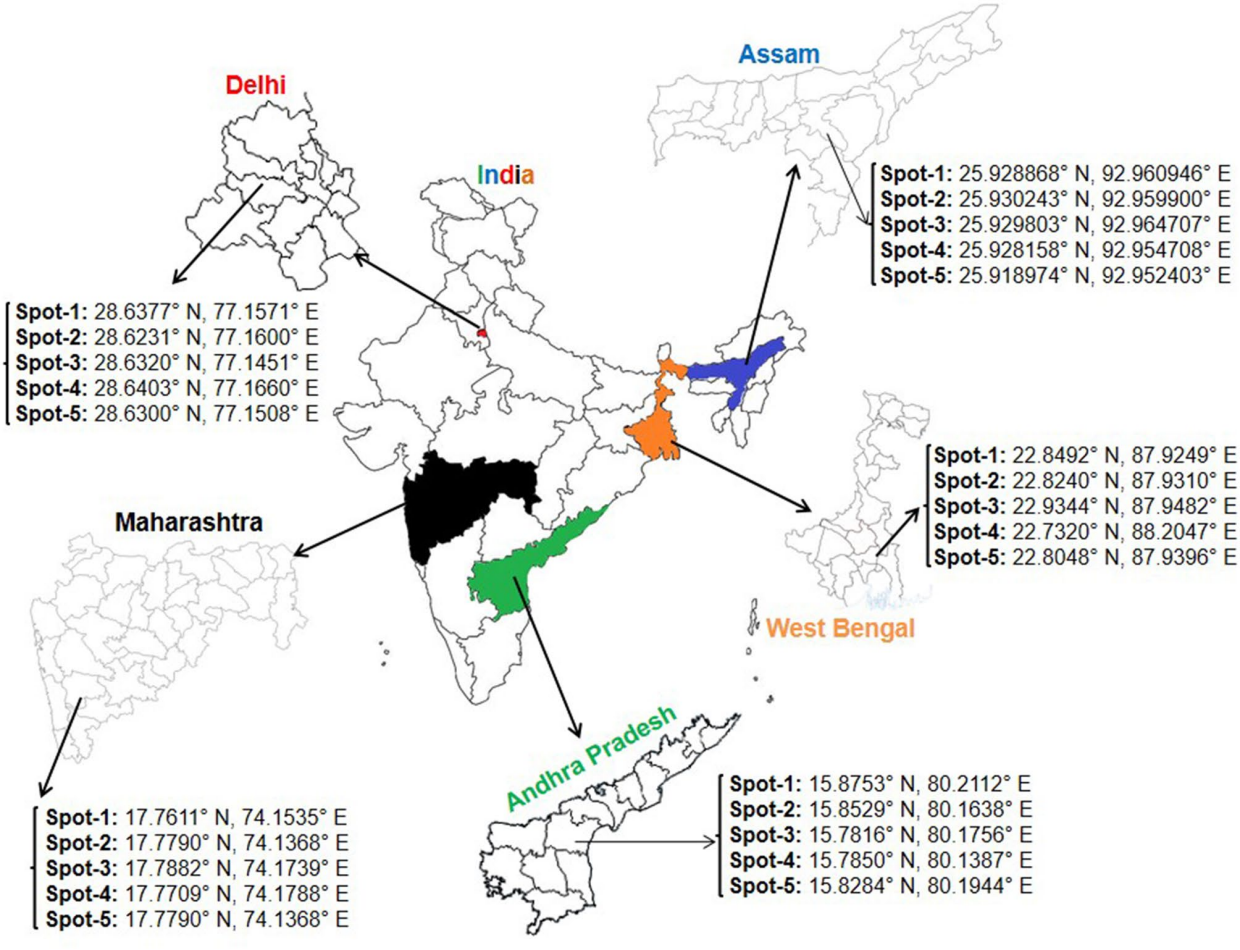
Figure depicted the map of India and the Global Positioning System (GPS) tracked location from where the *Murraya koenigii* leaf samples as well as soil samples have been collected. From every location samples are collected in quintuplet. The location were from southern, eastern, north eastern, northern and western part of India and the state were Andhra Pradesh (AP), West Bengal (WB), Assam (AS), Delhi (DL) and Maharashtra (MH) respectively with indicated GPS location. India-map and states map viz. AP, WB, AS, MH, DL were taken from google search (URL: https://in.pinterest.com) and further painted it to highlight the collection point.

The sites for sampling were selected to represent major *Murraya koenigii* grown areas and also variation in edaphic and climatic factors of plant growth. Accordingly, MK plants were collected from five different sites from each of 5 states of India namely Assam (North-East), West Bengal (East), Maharashtra (West), Andhra Pradesh (South) and Delhi (North)] depending on the geographical location. The sampling at five states across the different geographical location is shown in Fig. 1. The five different MK plants sample sites in a state were located within a distance of approximately 10 km from each other. The selected (unbiasedly) plants were 4–12 years old from their planting time. The exact location of each plant (collection site) was tracked by GPS and recorded. The different samples were collected by multiple collector in a way to accomplish the collection on a same day (15th Jan, 2019). For each plant sampled, leaves from the top shoots and soil samples from the trunk surrounding at a depth of 0–15 cm were collected and placed in a sterile zip lock bag with proper labeling. For the collection of soil samples an area was demarcated at a radius of 1 cm from the periphery of trunk and the composite soil was collected through sterilized trough from 0 to 15 cm depth and placed in a sterile zip bag. All the soil samples from different sites were collected in the same passion. The samples were brought to the laboratory within 24 h of collection from field. The soil samples were divided into two halves. First half was stored at 4 °C for determination of bacterial populations and the other half was shade dried for 5 days and stored for further analysis to determine different chemical parameters. The soil samples were marked as AP for Andhra Pradesh, MH for Maharashtra, DL for Delhi, AS for Assam and WB for West Bengal respectively as per their site of collection with five replication from 1 to 5.

### Weather parameter record collection

The detailed weather reports of the last 1 year (from February, 2018 to January, 2019) of selected sites were downloaded from the authenticated source, www.worldweatheronline.com site^20^. The different weather parameters such as the total rainfall, average humidity and average temperature were plotted to show the spatial and temporal variations.

### Soil microbial analysis

Soil rhizosphere microbiota plays a crucial role in formation of plant secondary metabolites and thereof monitoring plant’s immune system^21^. Total bacterial population in dry soil samples were determined by serial dilution pour plate method. Shade dried soil samples were carefully crushed individually in sterile motor and pestle into fine powdered and sieved through 100 µm sieve^22^. 1 g of the each finely sieved sample was taken and the serial dilution was performed from 10^3^ to 10^6^ in sterile HPLC grade water. From each serial diluted tube, 100 µL samples was plated separately on to nutrient agar plate and incubated for 24 h in 30 °C^22^. After incubation the microbial colonies were counted using colony counter. The plates containing 30–300 microbial population were taken for the CFU (Colony Forming Unit) calculation^23^.

### Analysis of soil physicochemical parameter

The physicochemical parameters such as pH and electrical conductivity (EC) of the shade-dried soil samples (sieved through a 0.5 mm sieve) were determined in 1:5 soil suspensions (w/v; soil/water) with Eutech digital pH meter and an EC meter respectively^24^. The organic carbon was determined by modified Walkley and black titrimetric method and the available phosphorus concentrations was determined in a UV–Vis Spectrophotometer (Shimadzu 1601, Japan) using the Mo–Sb colourimetric method^24,25^. Total nitrogen for soil samples was estimated by the method of Willits et al. using Kelplus-Classic DXVA automatic nitrogen analysis equipment^24^. Chloride concentrations were determined by titrimetric method^25^.

### Analysis of trace elements and micronutrients in soil samples

Trace element and/or micronutrient in soil samples were also determined in the shade-dried soil sieved through 0.5 mm pore diameter sieve. The concentrations of elements (As, Ca, Cd, Co, Cu, Fe, K, Mg, Mn, Na, Pb and Zn) were determined by an Atomic Absorption Spectrophotometer (AAS) (Shimadzu AA 7000) in the soil samples digested with 3:1 (v/v) HNO_3_ -HClO_4_ mixture^24^.

### Determination of moisture content in MK leaf biomass

1 g of fresh leaves of each sample was weighed separately and placed in marked glass petri dish at 80–90 °C at a hot air oven. Each plant leaf sample was dried in triplicate. Every alternative day, the samples were weighed and recorded until constant weight reached. The total moisture content was determined by subtracting the final weight from the compounding initial weight and expressed in percentage.

### Reagents

Methanol and water (HPLC grade), methanol (analytical grade), ethyl acetate, formic acid were purchased from Merck Millipore, USA. Analytical grade pre-coated silica gel chromatography plate was purchased from Merck Millipore, USA. All the chemicals used for the analysis of physicochemical and micronutrients parameters were purchased from Merck, India. The other reagents used in the study, were procured from Sigma Aldrich, USA.

### Solvent extraction of leaf and analysis

#### Extraction

Pre-weighed (100 g) washed leaves of each sample was separately ground in a mixer grinder with a pre-determined amount of methanol and soaked for 72 h to complete extraction^2^. The methanol extraction procedure was repeated for all the samples to confirm the completion of the extraction. The total methanol extract for each leaf sample were completely dried separately first in rotary evaporator (for the organic solvent) and latter in lyophilizer (for the aqueous part). Further, the methanolic extract for each sample was fractionated separately by EtOAc and water layering (70:30; v/v) and confirm the presence of mahanine in EtOAc fraction only (by spotting the EtOAc and water fraction with standard mahanine in TLC). The EtOAc fraction was dried in a rotary evaporator and stored in a vial at − 20 °C until further use. The yield of ethyl acetate fraction for each leaf sample was calculated.

#### HPLC analysis

High performance liquid Chromatography (HPLC) analysis was performed to quantify the mahanine content in the EtOAc fraction of the MK leaves of each sample separately. A standard curve was prepared with the standard mahanine isolated and purified in our previous study^2^ in a dose range from 0 to 500 µg/mL. The HPLC method that we have followed to run the standard compound and/or extract samples in this study was described by us previously^2^.

#### HPTLC analysis

High Performance Thin Layer Chromatography (HPTLC) was performed to understand the signature profiling of the phytochemicals in the MK leaves that were collected from five different zone of India. Single EtOAC fraction was randomly selected from each zone was used for spotting. A total of five EtOAc fractions (one from each zone), with standard mahanine were spotted in a pre-coated silica gel 60 F254 plates. The plates were developed in a solvent system containing chloroform: methanol (95:5) (v/v) + 0.1% formic acid (v/v) and visualized under 254 nm (in a TLC scanner).

### Statistical analysis

For comparison of a parameter between the two places the statistical analysis were performed by unpaired student t-test. All statistical tests were two-sided with 95% confidence interval (CI). A significance level was considered at *P* < 0.05. Statistical analyses were performed in GraphPad Prism 6.05 software.

## Results

### Analysis of total moisture content in fresh MK leaves

**Figure 2:**
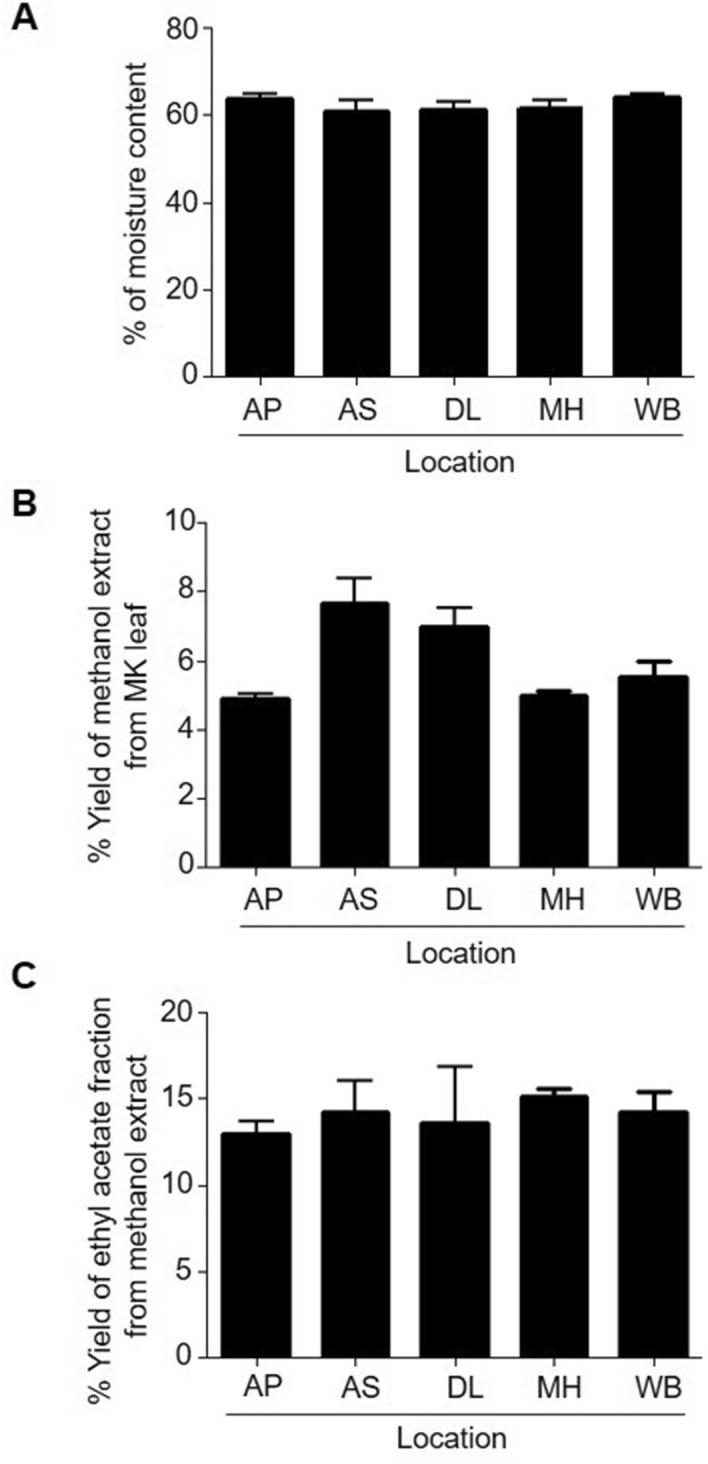
Analysis of extracted component: (**A**) total moisture content was measured in collected samples of MK leaves. The result represents the average of quintuplet samples with SD value from each location of AP, AS, DL, MH and WB respectively in %. (**B**) The total methanolic extract isolated from each samples are calculated (in %) and the graph represents the average of quintuplet samples with SD from each respective location. (**C**) The amount of ethyl acetate (EtOAc) fraction from the methanolic extract of each sample was calculated in % and the graph represents the average of quintuplet samples with SD value from each location.

Moisture content of a plant material affects the secondary metabolite extraction from plant parts^26,27^. As we have extracted the mahanine from the fresh leaves of different MK trees of varying location, it was necessary to check the moisture content of all the collected samples. The total average moisture content of the MK leaves varied from ~ 61 to 64% and did not vary significantly between locations (Fig. 2A). The moisture content was lowest (60.8 ± 2.9%) in the MK leaves collected from Assam (AS) state and highest (64.1 ± 0.7%) in MK leaves collected from West Bengal (WB) (Fig. 2A).

### Yield of the extract and quantification of mahanine content

**Figure 3:**
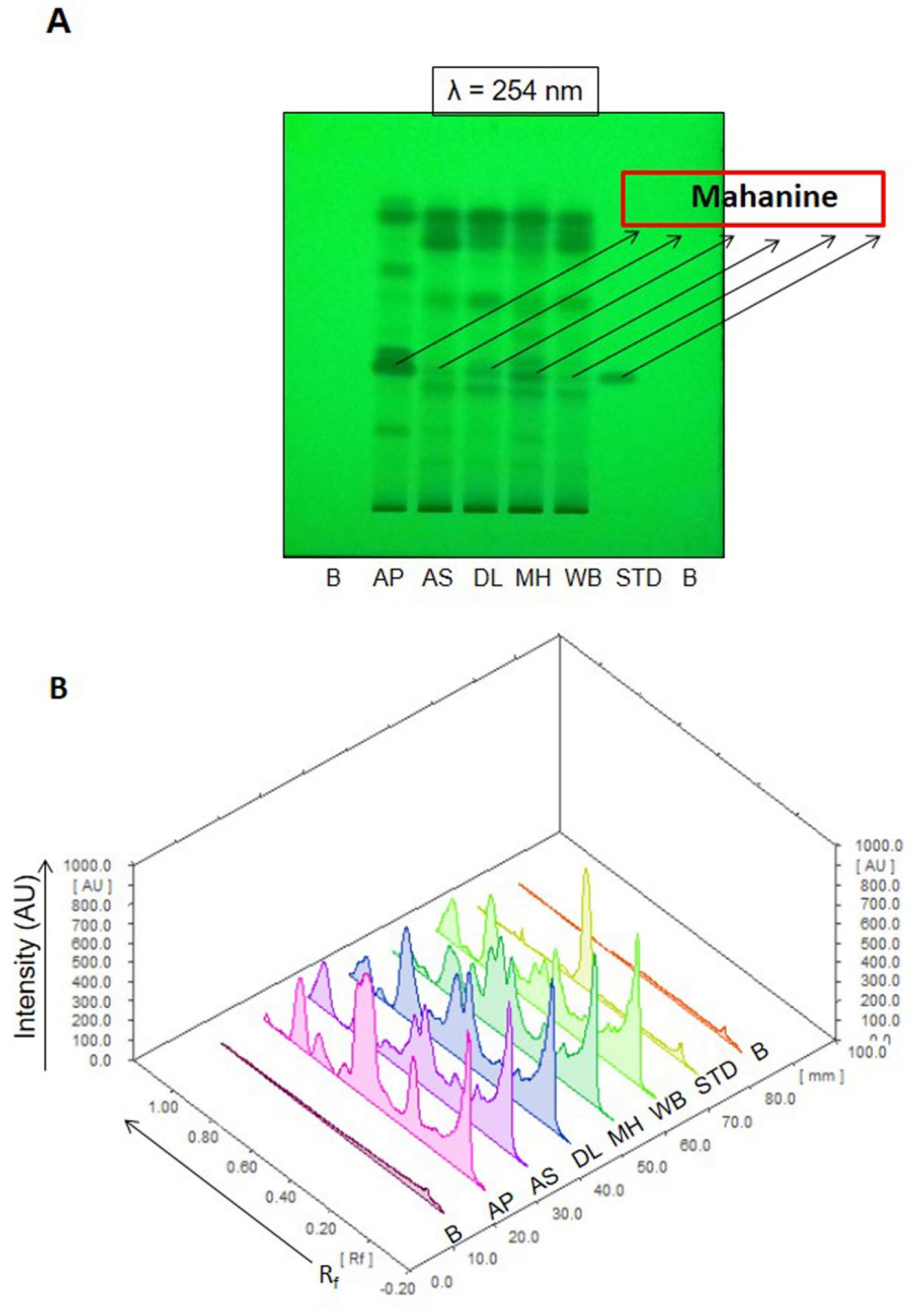
Chemical fingerprinting: (**A**) Thin Layer Chromatogram (TLC) showing the presence of mahanine with other molecules in EtOAc fraction of randomly selected one sample from each location. The plate was visualized under UV light at 254 nm and the plate was developed in a solvent system chloroform: methanol (95:5) (v/v) + 0.1% formic acid (v/v). (**B**) HPTLC fingerprinting profiling of EtOAC fraction of randomly selected one sample from each location. The plate was developed in a solvent system chloroform: methanol (95:5) (v/v) + 0.1% formic acid (v/v) and visualized under UV light at 254 nm. The TLC plate was analysed with a method, developed in winCATS (CAMAG, Switzerland, version: 1.4.8.2012) software.

**Figure 4:**
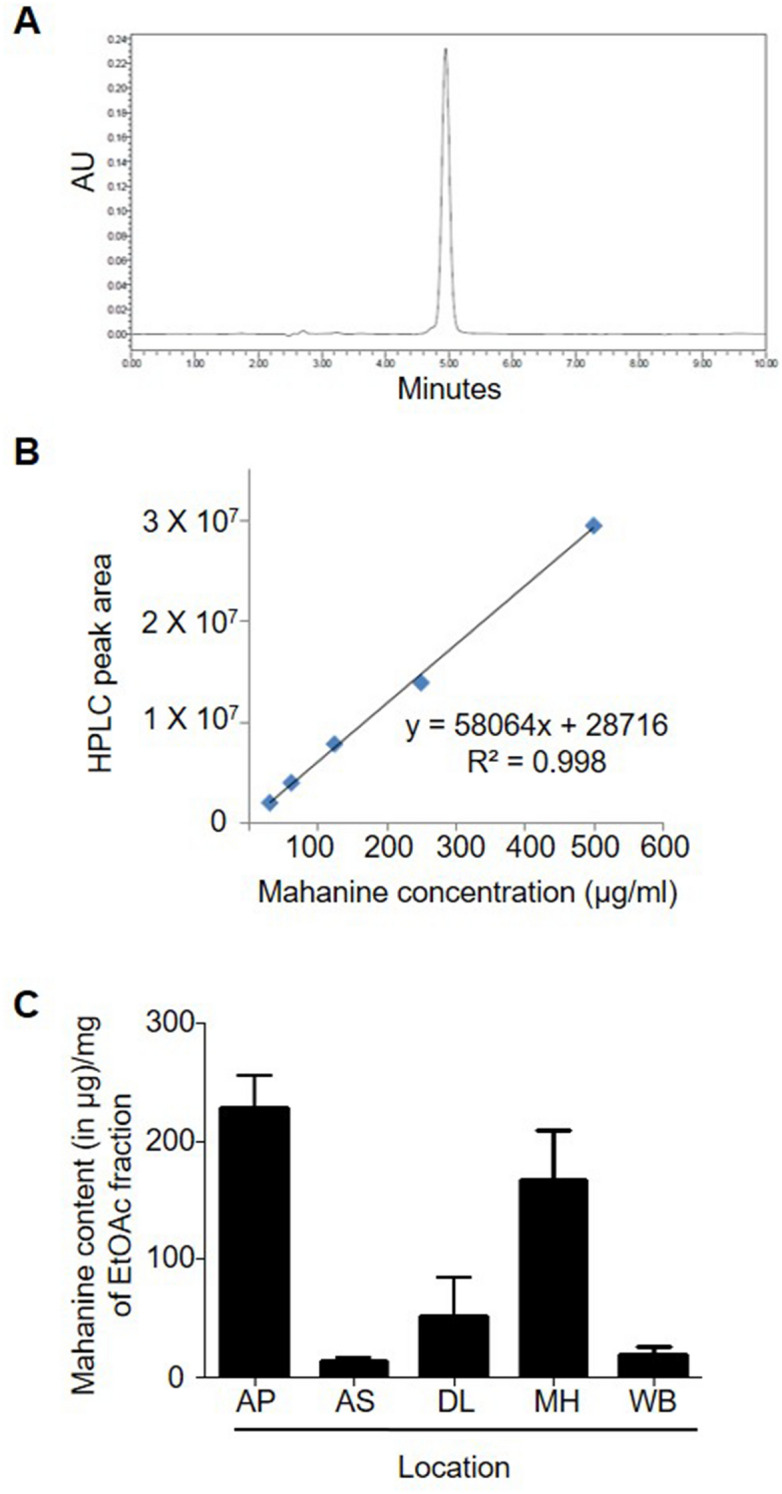
Quantification of mahanine: (**A**) HPLC profile of pure isolated mahanine. (**B**) The area under the corresponding peak are considered for a specific concentration of mahanine from HPLC method are used to draw the standard curve in a dose range from 0 to 500 µg/mL. (**C**) Quantification of mahanine (in microgram per mg) in EtOAC fraction of each collected samples. The graph represents the average quantity with SD of quintuplet samples from each location.

The complete methanol extract was separately lyophilized, weighed and the extract yield was determined for the leaves collected from different sites. The yield of methanolic extracts were significantly less in Andhra Pradesh (AP) and Maharashtra (MH) compared to that of Assam (AS) or Delhi (DL) (Fig. 2B). Percentage yield of methanol extract of the MK leaves collected from AP sites were found to be 4.9 ± 0.3%, whereas samples from AS site yielded 7.7 ± 1.6% (Fig. 2B). Further to quantify the yield of EtOAc fraction from methanolic extract, a known quantity of methanol extract was fractionated with EtOAc and water (70:30 v/v). MK leaves samples from MH (15.1 ± 0.9%), WB (14.3 ± 2.6%), DL (13.5 ± 7.4%) and AS (14.2 ± 4.0) yielded the highest amount of EtOAc fraction whereas AP samples yielded lowest amount (13.0 ± 1.6%) of EtOAc fraction per unit quantity of methanolic extract (Fig. 2C). However, no significant variation was observed in between the yield of EtOAc fractions of other sites (Figs. 2C, 3A,B). A standard curve was established using pure mahanine, ranges from 0 to 500 µg/mL using HPLC (Fig. 4A,B) to determine the mahanine content in EtOAc fractions of respective samples. Highest content of mahanine was found in samples collected from AP (228.5 ± 59.1 µg/mg) and the lowest was found in samples from AS (13.5 ± 5.4 µg/mg).The order of mahanine content in EtOAc fractions from different sites was AP > MH > DL > WB > AS (Fig. 4C).

### Detailed weather analyses of the past 1 year and correlation with mahanine content

Weather conditions and rain fall records of medicinal plant cultivation sites could be one of the key factors for development of secondary metabolites^26^. To assess the relation of these conditions in the formation of mahanine in MK leaves, a detailed scrutiny of the last year’s weather conditions at different sites were conducted. In AP and MH, where the mahanine content was maximum among the five selected locations, the average temperatures were 29.3 ± 2.6 °C and 25.2 ± 2.4 °C respectively for the 1 year preceding sample collection (Fig. S1A). The average humidity for those two sites were recorded as 67.7 ± 6.4 and 55.3 ± 21.1%, respectively (Fig. S1B). On the other hand, the sites AS, DL and WB (mahanine content of these sits were low) had the temperature record of 25.4 ± 2.6, 29 ± 6.3 and 28.7 ± 3.1 °C, respectively (Fig. S1A). Interestingly, the average temperature analyses showed, the more variance in temperature yielded less biosynthesis of mahanine in MK leaves. The record of average rain fall for these sites for the last 1 year showed that AS had ~ twofold higher rain fall than the highest mahanine yielding location AP (Fig. S1C) and the rainfall of low yielding site WB was not conspicuously different that of AP site. In the same time, the record of the approximate average age of the experimental plant had shown that DL has the higher approximate average age with compare to others. Altogether, the results concluded that the heavy rain fall and the age of the plant are considerable phenomena to producing the alkaloid metabolite like mahanine in the plant parts.

### Total micronutrient and heavy metal analysis in the soil samples and correlation with mahanine content

In the present study, soil of AP-1 site showed the highest potassium (K) content and MH-4 site & MH-5 site showed lowest potassium (K) content among 25 soil samples (Table 2). In case of Na, soil of AP-2 site showed highest concentration and soil of WB-1 site showed lowest concentration. The Ca and Mg concentration of soil of AS-4 site were significantly lower while soil of MH-3 site showed significantly high concentration of Ca and soil of MH-4 & AP-4 sites showed highest Mg concentration (Table 2). Among different heavy metals (Zn, Cu, Fe, Cd, Cr, Ni and Pb), the concentrations of Zn was highest in soil of MH-1 site and lowest in soil of AP-2 site and amount of Cu was highest in DL-4 site soil and lowest in MH-4 site soil (Table 1). Similarly, the concentration of Fe was highest in soil of MH-5 site and lowest in WB-1 site (Table 1). Concentration of Co and Mn was found to be highest in soil of MH-4 site and lowest in AS-4, DL-4, and DL-5 sites (Table 1). Cd was found to be below detection level in every site. Soil of DL-5 site has highest amount of Pb whereas AP-5 site soil had lowest amount. Concentration of arsenic (As) level was highest in soil of MH-1 site and lowest in soil of AS-5 site (Table 1).

**Table 1:** Details of concentration of heavy metal and salt in experimental soil sample from the selected origin.

Location	Sample Id	Concentration of heavy metal (mg/kg)							
		Cu	Pb	Co	Zn	Fe	Mn	Cd	As
Assam (AS)	AS-1	22.665	47.005	12.91	46.98	11,027.5	210.94	BDL	1749.69
	AS-2	22.385	63.52	22.845	59.78	15,410.8	538.59	BDL	2714.13
	AS-3	22.245	47.015	14.4	53.05	13,160	244.69	BDL	2220.66
	AS-4	20.005	53.355	13.655	37.18	10,074.8	85.21	BDL	2289.74
	AS-5	18.605	47	20.36	33.535	16,569.8	267.49	BDL	1737.45
Maharastra (MH)	MH-1	169.69	45.735	30.29	149	20,896.3	532.96	BDL	5062.5
	MH-2	155.84	47.005	25.82	90.37	21,643	408.19	BDL	4845.96
	MH-3	160.74	36.84	30.54	114.92	20,058.5	534.98	BDL	4845.96
	MH-4	170.81	50.815	34.015	103.17	20,955.3	606.08	BDL	4885.47
	MH-5	107.02	43.195	24.33	68.745	21,642.8	441.39	BDL	5170.5
Andhra Pradesh (AP)	AP-1	39.31	47.005	29.545	59.29	8457.5	343.36	BDL	2615.45
	AP-2	37.91	52.085	25.325	4.85	20,134	357.41	BDL	2309.49
	AP-3	65.47	49.545	23.59	120.78	14,313.8	232.74	BDL	1806.12
	AP-4	46.165	41.92	26.07	58.35	14,056.3	339.33	BDL	2210.81
	AP-5	21.965	34.3	26.32	34.6	13,299	266.21	BDL	3069.45
Delhi (DL)	DL-1	27.7	52.085	11.42	80.565	9389.8	222.43	BDL	3237.21
	DL-2	33.015	52.135	16.385	88.1	16,611	263.87	BDL	3207.6
	DL-3	32.595	62.25	11.67	101.83	8318.3	189.51	BDL	3266.82
	DL-4	27.4	33.03	9.685	124.9	8060.8	182.74	BDL	3108.92
	DL-5	35.675	80.035	9.685	111.7	12,206.5	291.21	BDL	3444.48
West Bengal (WB)	WB-1	14.83	47.005	11.67	59.78	8035	269.46	BDL	3168.14
	WB-2	28.96	40.65	10.18	121.66	9631.8	345.78	BDL	2931.26
	WB-3	18.185	50.815	10.43	107.74	8663.5	289.1	BDL	2802.96
	WB-4	21.965	45.735	15.64	104.74	12,645	402.25	BDL	2951.01
	WB-5	20.005	45.74	13.905	73.245	11,614.8	360.25	BDL	3503.7

*BDL* below detection limit.

### Detailed physicochemical analysis of the soil samples and correlation with mahanine content

The pH values were found to be highest in the soil of AP-2 site and lowest in the soil of AS-4 site (Table 2). The average pH of the soil samples from AS was calculated as 6.1 i.e., acidic soil environment for MK plant growth. The soil samples from AP and MH had the average pH of 8.8 and 8.4, respectively and indicates alkaline environment for MK plant growth (Table 2). The remaining soil samples of DL and WB had pH near neutral level. The electrical conductivity (EC) of different soil samples was found to be in the range of 54.8–694 µS/cm. The EC values were found to be highest for the soil of AP-4 site and lowest for the soil of AS-1 site (Table 2). The total organic carbon and available phosphorus of soil samples ranged from 0.744 to 2.16% and 0.5–2.3%, respectively (Table 2). Available nitrogen in soil samples ranged from 0.294 to 0.785% (Table 2).

**Table 2:** Details soil physiological parameters and important ion concentration in experimental soil sample from the selected origin.

Location	Sample Id	Parameters and unit						Concentration of ion (mg/kg)				
		Colour of the soil	pH	Conductivity µS/cm	Phosphate (mg per 100 g)	Organic carbon (%)	Total nitrogen (%)	Chloride	Ca	Mg	Na	K
Assam (AS)	AS-1	Pale brown	6.4	54.8	0.92	1.16	0.294	85.2	2.66	1	0.32	0.36
	AS-2	Pale brown	6.6	99.0	1.36	1.27	0.434	78.1	4.15	1.18	0.39	0.31
	AS-3	Pale brown	6.4	82.0	0.94	1.09	0.672	99.4	4.84	1.36	0.35	0.32
	AS-4	Pale brown	5.3	83.5	2.3	0.815	0.602	71	0.94	0.75	0.38	0.24
	AS-5	Reddish brown	5.9	72.1	1.96	1.20	0.743	78.1	0.54	0.59	0.42	0.33
Maharastra (MH)	MH-1	Blackish brown	8.5	302	1.22	1.70	0.336	177.5	48.44	1.74	1.03	0.12
	MH-2	Brown	8.6	238	1.64	1.34	0.378	134.9	45.05	1.68	1.51	0.1
	MH-3	Blackish brown	8.5	210	1.14	1.20	0.532	149.1	49.68	1.71	1.54	0.1
	MH-4	Brown	7.9	98.0	1.32	1.80	0.602	142	20.78	1.68	0.75	0.09
	MH-5	Blackish brown	8.5	306	1.02	1.06	0.480	156.2	45.37	1.68	0.79	0.09
Andhra Pradesh (AP)	AP-1	Blackish brown	8.5	209	0.78	0.815	0.757	85.2	42.03	1.72	0.5	1.01
	AP-2	Blackish brown	9.5	456	0.66	0.779	0.434	113.6	28.87	1.73	2.09	0.66
	AP-3	Brown	9.4	249	0.5	1.34	0.574	78.1	40.71	1.66	0.72	0.2
	AP-4	Brown	8.25	694	0.82	0.744	0.785	99.4	40.82	1.74	1.35	0.58
	AP-5	Blackish brown	8.55	554	0.48	0.673	0.574	99.4	22.47	1.67	0.76	0.54
Delhi (DL)	DL-1	Brown	7.3	191	1.28	2.16	0.438	220.1	17.18	1.57	0.27	0.27
	DL-2	Pale brown	8.5	245	0.6	1.84	0.520	205.9	27.07	1.7	0.38	0.54
	DL-3	Brown	7.7	136	0.88	1.41	0.650	184.6	18.3	1.54	0.26	0.21
	DL-4	Dark brown	8.0	181	0.64	1.41	0.490	255.6	12.7	1.57	0.25	0.22
	DL-5	Blackish brown	8.3	191	1.14	1.80	0.632	227.2	34.37	1.65	0.29	0.25
West Bengal (WB)	WB-1	Brown	6.9	186	0.54	1.70	0.434	177.5	2.04	1.09	0.23	0.19
	WB-2	Pale brown	5.5	459	0.72	1.77	0.855	170.4	5.14	1.3	0.24	0.26
	WB-3	Dark brown	7.3	133	0.58	1.70	0.504	184.6	6.22	1.2	0.24	0.2
	WB-4	Brown	8.3	146	0.7	1.66	0.715	170.4	4.5	1.5	0.41	0.35
	WB-5	Brown	7.1	606	1.92	1.09	0.532	163.3	5.11	1.34	0.42	0.25

### Microbial analysis of the soil samples and correlation with mahanine content

Based on observation of morphology of the bacterial colony, five different types of bacterial colonies were detected in the soil samples. The microbial plates that contains a minimum of 30–300 bacterial colonies were only considered and counted^23,24^. From the experimental data, no significant difference in the CFU was observed among all the soil samples tested from different locations. But, a trend of slightly high bacterial population (CFU) was observed in soil samples collected from AP and MH (6.7 ± 0.3 and 6.9 ± 0.3 log CFU) compared to other locations like AS and WB (6.5 ± 0.3 and 6.6 ± 0.3 log CFU) (Table 3). The soils of AP and MH sites showed five diverse bacterial populations, whereas soil of AS sites contained only three types of bacterial colonies (Fig. S1F).

**Table 3:** Total microbial CFU in experimental soil sample.

Sample	Log cfu/g					
ID	01	02	03	04	05	Mean ± SEM
AS	7.48 ± 0.17	7.06 ± 0.43	6.15 ± 0.10	5.89 ± 0.28	5.98 ± 0.07	6.51 ± 0.32
AP	7.51 ± 0.21	7.19 ± 0.29	6.35 ± 0.14	5.82 ± 0.07	6.62 ± 0.20	6.70 ± 0.30
WB	7.45 ± 0.21	6.84 ± 0.43	6.10 ± 0.06	5.74 ± 0.12	6.44 ± 0.09	6.55 ± 0.28
MH	7.58 ± 0.23	7.48 ± 0.44	6.27 ± 0.06	5.89 ± 0.04	6.92 ± 0.17	6.86 ± 0.31
DL	7.46 ± 0.12	7.33 ± 0.48	6.16 ± 0.09	6.05 ± 0.0.09	6.83 ± 0.19	6.71 ± 0.33

## Discussion

The accumulation and/or formation of secondary plant metabolites helps the plants in germination, growth and survival from different perspectives^27^; and those plant secondary metabolites have been the rich sources for the drug development research for various aliments since centuries^28^. Mahanine, a carbazole alkaloid isolated from the *Murraya koenigii* plant parts has been reported as important bioactive molecule for different health disorder like cancer and diabetes^2, 7^. From our previous research findings, we have established for the first time that, mahanine is a potential drug candidate to treat breast cancer in subtype regardless manner^2^. During the isolation procedure of mahanine from MK leaves in our preceding study, we have observed a peculiar phenomenon that the yield of mahanine was significantly varied based on the geographical location of the plant sample collection. The present study has provided interesting perspectives on mahanine content in MK leaf that were collected from different geographical locations of India and along with the role of soil and weather conditions of those sites towards this phenomena. Mahanine content in MK leaves collected from AP sites were ~ 16.9 times higher and MH sites were ~ 12.4 times higher compared to that of MK leaves collected from AS sites. The average pH of the soil samples of AP and MH sites were found to be alkaline in nature (pH 8.8 and 8.4 respectively), whereas soil samples from AS sites were acidic in nature (pH 6.1). These finding suggests that soil alkalinity plays a significant role in the accumulation of higher level of mahanine in MK leaf. Accordingly, the electric conductivity (EC) of the soil samples also plays a significant role in formation of mahanine in MK leaves; plant mahanine content is directly proportional to electric conductivity. The mean EC of AP site (highest mahanine content) soils were 432 µS/cm, whereas for AS site (lowest mahanine content) soils were 78.3 µS/cm. Assam site has two times higher average rain fall than the AP sites. Higher rainfall leach down soil basic cations and make soil acidic and also it cause leaching of soluble salts down the soil profile reducing EC values. The high salinity of soil sample have crucial role in plant secondary metabolite formation^29^. MH sites, which are in a similar geographical region as the AP sites also had high soil pH and EC which in turn showed high mahanine content in MK leaf. The soil microbiota also plays a crucial role in plant defense system and directly correlated with plant secondary metabolite formation^23^. Soil microbial analysis of this study revealed that more the value of log CFU of bacteria, greater was the concentration of mahanine in MK leaves. Interestingly, more diverse microbial population of bacteria was found in soil samples collected from AP sites, where the MK leaf mahanine content was highest compared to that of AS sites, where lowest soil microbial population as well as mahanine content. Although, single plate count microbial assay does not provide entire range of soil bacterial diversity, there was an indication of relation between bacterial diversity and mahanine content. A detailed culture independent mechanistic bacterial diversity study can depicts the role of microbe(s) in secondary metabolite (in specific mahanine) formation in MK leaves need to be investigated. Other micronutrient of soil such as Na, K, Ca and Mg are also reported to play important role in formation of specific type of plant secondary metabolites^21^. These nutrients in soils of AP and MH sites were found significantly higher amount compared to those of WB or AS sites. The other factors such as temperature, humidity and plant’s moisture content were found to have no significant relation with the mahanine content of MK leaf. Altogether, the present study provides the evidence of variation in mahanine in MK leaves depending upon the soil physicochemical and biological parameters, the direct and interactive contribution of each factors cannot be accounted from this data. This study warrant for research under controlled environment to establish quantitative relationship between different weather and soil parameters and mahanine content in MK leaf to develop agro-technology in enriching a specific bioactive metabolite.

## Supplementary information

Supplementary Information.

## Data Availability

All data generated or analysed during this study are included in this published article (and its Supplementary Information files).

## Abbreviations

MK: *Murraya koenigii*
CFU: Colony Forming Unit
HPLC: High Performance Liquid Chromatography
HPTLC: High Performance Thin Layer Chromatography
EC: Electric conductivity
AP: Andhra Pradesh
WB: West Bengal
AS: Assam
DL: Delhi
MH: Maharashtra
