# Supplementary material for: Variation in biosynthesis of an effective anticancer secondary metabolite, mahanine in Murraya koenigii, conditional on soil physicochemistry and weather suitability
Source: Sci Rep. 2020 Nov 18;10:20096. doi: 10.1038/s41598-020-77113-y (PMC7675983; doi:10.1038/s41598-020-77113-y)
Supplement: Supplementary file 1 — Supplementary Information. [file 41598_2020_77113_MOESM1_ESM.docx]

**Variation in biosynthesis of an effective anticancer secondary metabolite, mahanine in *Murraya koenigii*, conditional on soil physicochemistry and weather suitability**

Raghuram Kandimalla^1,2,$^, Momita Das^1,$^, Sagar R Barge^1^, Partha Pratim Sarma^1^, Dibya Jyoti Koiri^3^, Arundhuti Devi^3^, Arjun Kumar Karki^1^, Anil Kumar^1^, Rajlakshmi Devi^1^, Bikas C Pal^1^, Narayan C Talukdar*^1^ and Suman Kumar Samanta*^1^

^$^Contributed equally

**Affiliation of authors:**

1. **Drug Discovery Laboratory, Life Sciences Division, Institute of Advanced Study in Science and Technology, Vigyan Path, Paschim Boragaon, Guwahati-781035, Assam, India.**
2. **Current address: James Graham Brown Cancer center, University of Louisville, Louisville, KY-40202.**
3. **Environmental Chemistry Laboratory, Resource Management and Environment Section, Life Sciences Division, Institute of Advanced Study in Science and Technology, Vigyan Path, Paschim Boragaon, Guwahati-781035, Assam, India.**

**^*^To whom correspondence should be addressed:**

1. Suman Kumar Samanta, **Drug Discovery Laboratory, Life Sciences Division, Institute of Advanced Study in Science and Technology, Vigyan Path, Paschim Boragaon, Guwahati-781035, Assam, India. Email:** [**sumansamanta699@gmail.com**](mailto:sumansamanta699@gmail.com)**;** [**skshamanta@iasst.gov.in**](mailto:skshamanta@iasst.gov.in)**; Phone:** 0361227361
2. Narayan C Talukdar, **Drug Discovery Laboratory, Life Sciences Division, Institute of Advanced Study in Science and Technology, Vigyan Path, Paschim Boragaon, Guwahati-781035, Assam, India. Email:** nctalukdar@yahoo.com**; Phone:** 0361227361

**Fig S1:** Weather and Plant physiology analysis: Average (A) temperature variation, (B) humidity and (C) rainfall was noted and graphed from the [www.worldweatheronline.com](http://www.worldweatheronline.com) for the selected location for the last one year from the sample collection date. (D) The graph represents the average height (in feet) of the plant of a selected location (E) the graph represents the average age (in years) of the plant of a selected location. (F) Representative figure of agar plate showing the variation after forming the microbial growth from the sample of AS and AP respectively.


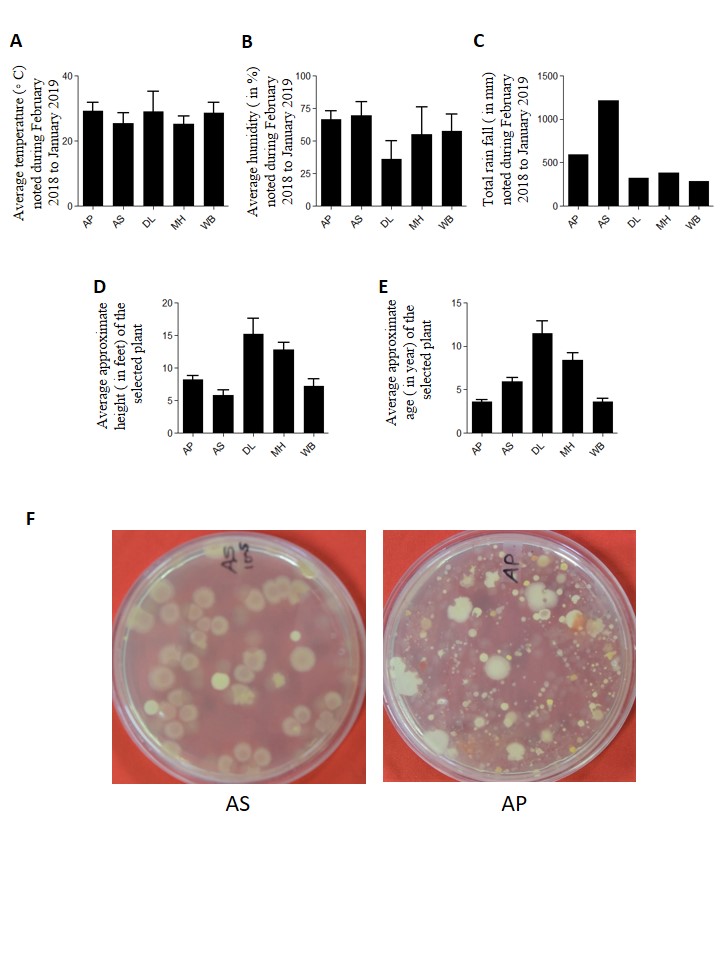


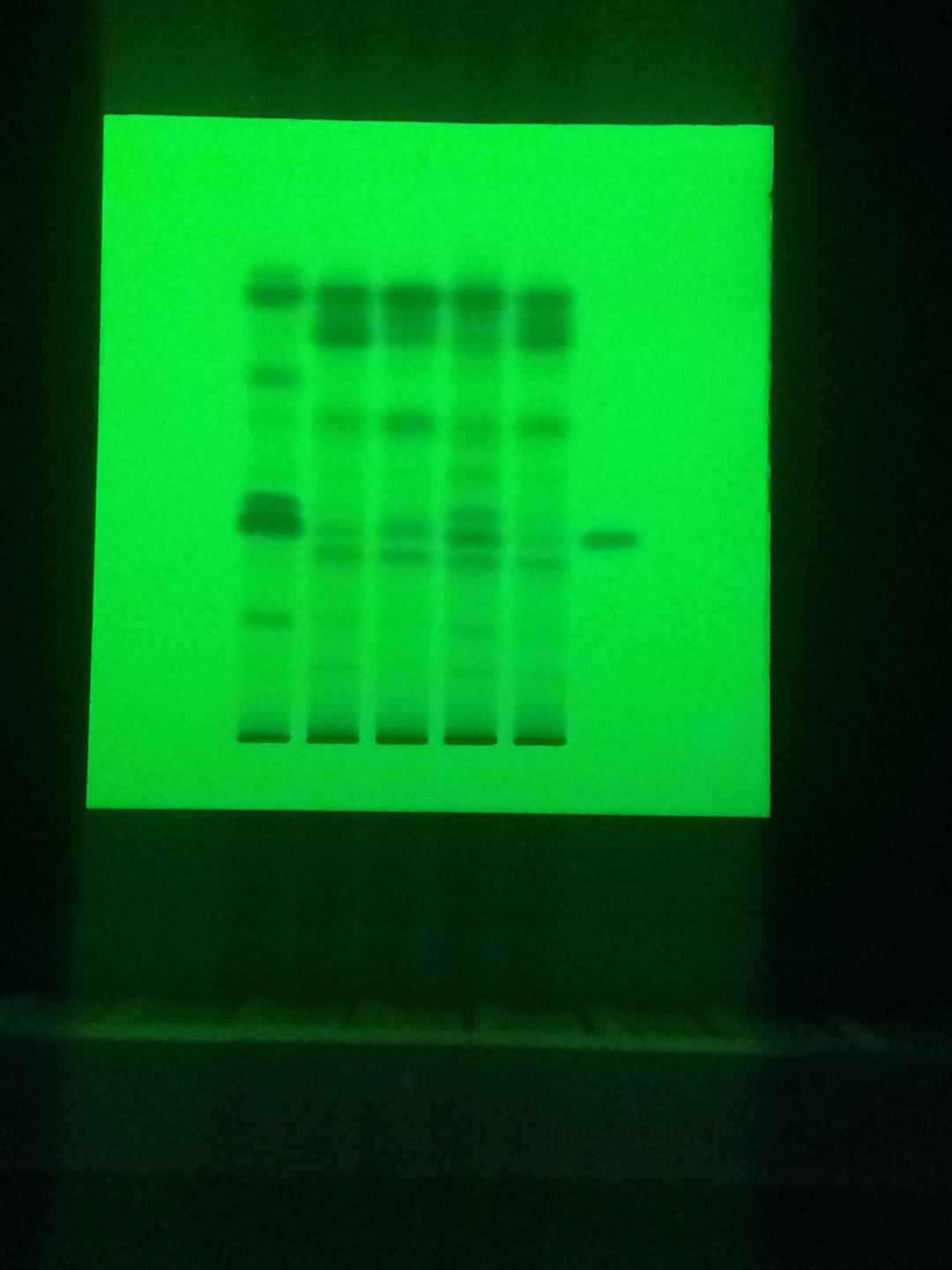


B AP AS DL MH WB STD B

Fig 4A: Total raw image without any cropping.
